# Body talk and the internalization of the ideal body image: examination based on the tripartite influence model

**DOI:** 10.1186/s40337-025-01444-2

**Published:** 2025-11-13

**Authors:** Nahori Ito, Akira Hasegawa, Masaki Adachi, Shin-ichi Oura, Tetsuya Yamamoto, Yuko Matsuda, Takuro Tomita

**Affiliations:** 1 Graduate School of Health and Welfare Sciences, International University of Health and Welfare, 4-1-26 Akasaka, Minato-ku, Tokyo, 107-8402 Japan; 2 Department of Psychology, International University of Health and Welfare, 4-1-26 Akasaka, Minato-ku, Tokyo, 107-8402 Japan; 3 Department of Psychology, Meiji Gakuin University, 1-2-37 Shirokanedai Minato-ku, Tokyo, 108-8636 Japan; 4 Faculty of Human Relations, Tokai Gakuin University, 5-68 Naka-kirino, Kakamigahara, 504-8511 Gifu Japan; 5 Graduate School of Technology, Industrial and Social Sciences, Tokushima University, 1-1, Minamijosanjima-cho, Tokushima, 770-8502 Japan; 6 Institute of Human Sciences, University of Tsukuba, 1-1-1 Tennodai, Tsukuba, 305-0006 Ibaraki Japan; 7 Faculty of Letters, Chuo University, 742-1 Higashinakano Hachioji-shi, Tokyo, 192-0393 Japan

**Keywords:** Body talk, Fat talk, Muscle talk, Body dissatisfaction, Body image

## Abstract

**Background:**

Based on the tripartite influence model, this longitudinal study investigated the relationship between body talk and the internalization of ideal body images, mainly focusing on female university students. It was hypothesized that negative fat talk and positive body talk would interact with exposure to thin-ideal images on the internet to influence the internalization of the thin ideal, leading to body fat dissatisfaction among women. This study also tested the hypothesis that negative muscle talk—a form of body talk focusing on dissatisfaction with one’s muscularity—would be associated with the internalization of athletes’ bodies, which corresponds to a toned or mesomorphic physique, among women.

**Methods:**

We conducted a two-wave longitudinal study over a 4-week interval with female (*n* = 386, mean age = 19.22 years) and male students (*n* = 216, mean age = 19.62 years) who completed self-report measures assessing frequency of body talk, the internalization of the thin ideal or athletes’ bodies, exposure to thin-ideal images on the internet, and body dissatisfaction.

**Results:**

Path analyses conducted separately by gender revealed no significant interaction between baseline body talk and exposure to thin-ideal images in predicting the internalization of the thin ideal four weeks later. However, exposure to thin-ideal images directly predicted increased internalization of the thin ideal for both genders after controlling for baseline levels. Exposure to thin-ideal images indirectly predicted body fat dissatisfaction through increased thin-ideal internalization among women. Additionally, baseline negative muscle talk was associated with increased internalization of athletes’ bodies for women only. Among men, baseline negative fat talk was positively related to the subsequent internalization of athletes’ bodies.

**Conclusions:**

Negative fat talk and positive body talk did not moderate the effect of exposure to thin-ideal images on the internalization of the thin ideal. In contrast, exposure to thin-ideal images emerged as a significant factor in promoting the internalization of the thin ideal. Among women, negative muscle talk promoted the internalization of athletes’ bodies. These findings may advance future research in eating disorder pathology and media psychology.

**Supplementary Information:**

The online version contains supplementary material available at 10.1186/s40337-025-01444-2.

## Abstract (plain language summary)

This study focused on the influence of three types of body talk: negative fat talk, which involves statements like “I feel so fat” to express dissatisfaction with body fat; negative muscle talk, reflecting frustration about not being muscular enough; and positive body talk, which reflects acceptance of one’s body and related lifestyle habits. A survey of 602 university students indicated that whether women engaged in negative fat talk was not associated with a subsequent increase in the internalization of the culturally promoted thin ideal (thin-ideal internalization) four weeks later. In contrast, women who were exposed to more thin-ideal depictions of the female body online were more likely to have greater thin-ideal internalization, which in turn increased their body fat dissatisfaction. Additionally, women who engaged more in negative muscle talk showed a greater increase in the internalization of athletes’ bodies, that is, a toned or muscular physique. Unlike women, men who engaged more in negative fat talk showed a greater increase in the internalization of athletes’ bodies. These findings revealed that, in addition to exposure to thin-ideal depictions, long known to increase body image concerns, engaging in body talk also contributes to these concerns.

**Supplementary Information:**

The online version contains supplementary material available at 10.1186/s40337-025-01444-2.

## Background

### Body talk

Body talk refers to how individuals communicate about their bodies with others and is categorized into three distinct types: negative fat talk, negative muscle talk, and positive body talk [1]. Negative fat talk involves expressing negative sentiments about one’s weight, exemplified by statements such as “I’m so fat.” This type of discourse is particularly prevalent among women [2]. Negative muscle talk, characterized by a negative tone, focuses on concerns about muscularity, including the desire or perceived pressure to build more muscles, as expressed in statements such as, “I wish I were more muscular” [1, 3]. In contrast, positive body talk encompasses conversations with affirming and constructive content, such as expressing self-acceptance, affirming one’s body, and discussions promoting healthy eating habits [1]. Each type of body talk has been identified as a potential determinant of body fat dissatisfaction, which is the subjective evaluation of one’s body shape as excessively fat and the perceived discrepancy between one’s actual and ideal body image [4], and muscle dissatisfaction which refers to the subjective evaluation of one’s body shape as lacking muscularity or tone [5, 6].

Guided by the tripartite influence model and recent findings, this study examined three hypotheses in a sample of female university students: (a) women who more frequently engage in negative fat talk would exhibit a more substantial indirect effect of exposure to thin-ideal images on body fat dissatisfaction through the internalization of the thin ideal, (b) women who more frequently engage in positive body talk would experience a weaker indirect effect of such exposure on body fat dissatisfaction via the internalization of the thin ideal, and (c) negative muscle talk would be positively associated with the internalization of the athletes’ bodies. The following sections provide an overview of the tripartite influence model, its relationship with body talk, and the purpose of this study.

### Tripartite influence model

The tripartite influence model [7] is a foundational framework explaining how societal pressures to conform to thin-ideal body standards contribute to increased body fat dissatisfaction and the development of eating disorder symptoms. This model identifies three primary sources of pressure—family, peers, and media. Family and peer pressures often include teasing or negative feedback about one’s body, such as comments about weight [8, 9]. In contrast, media pressures initially stemmed from portrayals of thin body shapes and advice on achieving such figures on television and in magazines [9]. With the advent of the internet, social networking sites like Facebook have become additional sources of media-related pressure on body image [10].

These pressures reinforce culturally accepted appearance ideals, such as thinness for women [11], and perpetuate the internalization of the thin ideal [12]. The internalization of the thin ideal refers to the degree to which an individual cognitively accepts societal norms regarding appearance or engages in behaviors to align with these standards [13]. Studies have identified internalization as a key predictor of body dissatisfaction, which elevates the risk of developing eating disorder symptoms [14].

A recent meta-analysis reported that the internalization of the thin ideal is positively associated with body dissatisfaction across gender, age, ethnicity, and BMI, supporting the tripartite influence model [15]. Furthermore, studies show that all three sources of pressure—family, peers, and media—are associated with increased internalization of the thin ideal. For example, research on adolescent girls has demonstrated that family and peer pressures are linked to heightened internalization of the thin ideal [16, 17]. A multi-country study of girls aged 12–18 years in Australia, China, India, and Iran found that family pressures were consistently associated with internalization of the thin ideal across all countries. In contrast, peer pressures were significant in Australian and Indian samples [18]. Media pressures, including those from traditional and social media, have been shown to significantly predict internalization of the thin ideal, even after accounting for family and peer pressures [17]. Meta-analytic findings further highlight that social networking site usage is associated with internalization of the thin ideal, with photo-related activities having a more substantial impact than general social networking site use [19, 20].

Tylka [21] extended the tripartite influence model to the ideal body image of being muscular with no fat that men often idealize. This body shape that men tend to idealize is referred to as a *mesomorphic body*. Tylka’s extended model, which focuses on men’s ideal body shapes and body dissatisfaction, posits that media, peer, family, and romantic partner pressures promote the internalization of a mesomorphic ideal, contributing to both muscle and fat dissatisfaction. Tylka’s study [21] of male undergraduate students revealed that family and media pressures were significantly associated with higher internalization of the mesomorphic ideal, which in turn predicted increased muscle and body fat dissatisfaction. Previous studies [22, 23] have assessed the internalization of the mesomorphic ideal using the internalization-athlete subscale of the Sociocultural Attitudes Towards Appearance Questionnaire–3 [13] and its subsequent versions of the subscale [11] among women and men. In these studies, researchers have generally assumed that an athlete’s body represents the culturally idealized muscular physique, characterized by high muscle tone and minimal body fat.^1^

Schaefer et al. [11] point out that the media, family, and peers are pressured to adhere to different culturally sanctioned appearance ideals, which are a thin ideal body for women and a muscular ideal body for men. The evidence supports the notion that women tend to desire thinness, and men are likelier than women to desire a muscular body [24, 25]. However, recent trends suggest that women increasingly desire a toned, muscular-yet-thin body shape, influenced by the popularity of online “*fitspiration*” content, although this ideal differs from the mesomorphic body type often sought by men [6, 26]. Therefore, the emphasis on internalizing a muscular body shape, as outlined in the extended version of the tripartite influence model [21], is likely applicable to women as well, although their ideal is typically a toned, muscular-yet-thin body shape.

### Body talk based on the tripartite influence model

Stice et al. [27] proposed that peer pressure, which is assumed to contribute to body fat dissatisfaction in the tripartite influence model [7], encompasses conversation partners’ comments expressing dissatisfaction with their own weight and suggesting weight loss through exercise and dieting. Such comments are regarded as negative fat talk. In line with the assumptions of this model, Stice et al. [27] conducted an experimental study with female undergraduate students and demonstrated that exposure to negative fat talk led to increased body fat dissatisfaction.

Recent studies have suggested that a person’s *engagement in* negative fat talk mediates the relationship between *hearing* negative fat talk and internalization of the thin ideal and body fat dissatisfaction. Experimental studies conducted with female participants have demonstrated that participants exposed to negative fat talk were more likely to reciprocate with similar statements compared to those exposed to other types of conversations, such as discussions about clothing or positive body talk [28, 29]. This reciprocal behavior may be driven by underlying social motivations, as prior research [30] shows that individuals often mirror negative fat talk when exposed to it to foster a sense of connection and belonging within their social group.^2^ Furthermore, experimental studies [28, 29] have suggested that engaging in negative fat talk, rather than merely hearing it, is associated with increased body dissatisfaction. These findings can be interpreted to mean that engaging in negative fat talk rather than hearing it fosters cognitive processing that internalizes the thin ideal and, in turn, increases body dissatisfaction. Thus, the tripartite influence model can be extended to propose that peer pressure may lead individuals to engage in negative fat talk, which in turn reinforces the internalization of the thin ideal and increases body fat dissatisfaction.

In contrast, positive body talk is a counterpoint to negative fat talk and is hypothesized to have opposing effects [31]. Positive body talk promotes a favorable perception of one’s body and diminishes the importance of physical appearance by emphasizing self-acceptance and healthy behaviors. This form of discourse is thought to disrupt cognitive processes that cause the internalization of the thin ideal and mitigate body dissatisfaction.

Empirical research supports these assumptions. Cross-sectional studies indicate that negative fat talk is associated with heightened internalization of the thin ideal for women and men [24, 32, 33], increased body dissatisfaction for women [34, 35], unhealthy eating behaviors, and eating disorder symptoms among women [36, 37]. These studies also include findings that show a significant association between negative fat talk and increased body dissatisfaction for both women and men [24, 32, 33]. Longitudinal studies similarly suggest that negative fat talk decreases body satisfaction, as demonstrated in studies controlling for sex [38], and increases the discrepancy between actual and ideal body images over time, as shown in studies conducted exclusively with female participants [24].

Conversely, previous studies have shown that positive body talk is concurrently associated with decreased body dissatisfaction, with this relationship demonstrated for women in a study including both female and male participants [24], and for both women and men in another study [32]. In addition, a four-week longitudinal study in women showed that positive body talk was linked to reduced discrepancy between ideal and actual body image four weeks later [24]. The same study found no significant correlation between positive body talk and the internalization of the thin ideal for either women or men.

Negative muscle talk may influence body image through mechanisms similar to those of negative fat talk. These similarities can be explained by the shared negative content inherent in both types of body talk [1], as well as by theoretical explanations regarding dissatisfaction with body fat and muscles, both of which involve the internalization of an ideal body shape [7, 21]. Just as negative fat talk is thought to foster cognitive processing that internalizes the thin ideal, a notion supported by experimental findings [28, 29], negative muscle talk may similarly promote cognitive processing focused on achieving an ideal toned or mesomorphic physique, potentially leading to increased dissatisfaction with body fat and muscles.

Past research has found a positive correlation between negative muscle talk and the internalization of the athletes’ bodies—representing toned or mesomorphic body standards—among women and men [24], supporting this notion. Ito et al. [24] found that negative muscle talk was positively associated with body fat and muscle dissatisfaction in women. Their study revealed that negative muscle talk was associated with muscle dissatisfaction, but not with body fat dissatisfaction, among men. However, the same study reported no significant longitudinal associations between negative muscle talk and body fat dissatisfaction or body image discrepancy in either gender after four weeks.

### Limitations of previous studies

The studies reviewed above have several limitations. First, no longitudinal research has examined specific associations among women predicted by the tripartite influence model and recent studies. These include (a) the mediating role of the internalization of the thin ideal in the relationships between negative fat talk or positive body talk and body dissatisfaction and (b) the interaction between negative fat talk or positive body talk and exposure to thin-ideal body images on the internet in predicting the internalization of the thin ideal. Considering the tripartite influence model [7] and subsequent experimental findings [28, 29] described above, engaging in negative fat talk may intensify the cognitive processing of thin-ideal body images. Experimental evidence indicates that individuals who engaged in negative fat talk experience greater body fat dissatisfaction after viewing images of thin actresses or models compared to those who did not engage in negative fat talk [28, 29]. Thus, negative fat talk may elaborate cognitive processing activated by exposure to images of slim women. Conversely, positive body talk may disrupt these cognitive processes, reducing the internalization of the thin ideal when individuals view such images.

Second, previous research has not investigated the longitudinal associations between negative muscle talk and the internalization of a toned or mesomorphic body among women and men. Given the previously described similarities between negative fat talk and negative muscle talk [1], and the theoretical background associated with both [7, 21], negative muscle talk may similarly reinforce the internalization of a toned or mesomorphic body. Cross-sectional evidence supports this association, indicating a positive correlation between negative muscle talk and internalization of the athletes’ bodies, corresponding to the toned or mesomorphic physique, in both women and men [24]. However, longitudinal studies have not tested these associations. Longitudinal research is needed to establish temporal relationships between them.

### Purpose

This four-week longitudinal study addressed unresolved issues concerning body talk and the tripartite influence model. The current study primarily focused on the determinants of body fat dissatisfaction among female university students. Therefore, we tested the following hypotheses using a sample of females. The study had two primary objectives: (1) To examine whether the indirect effect of exposure to thin-ideal body images on the internet on body fat dissatisfaction, mediated by the internalization of the thin ideal, was moderated by the frequency of negative fat talk or positive body talk; (2) To determine whether negative muscle talk was associated with subsequent internalization of athletes’ bodies.

Previous research has highlighted gender differences in ideal body preferences, such as thinness for women and muscularity for men, as well as variations in types of body dissatisfaction, including body fat dissatisfaction and muscle dissatisfaction [25, 39]. Longitudinal evidence also suggests that the relationships between body talk and body dissatisfaction differ by gender [24]. Based on these findings, the current study conducted separate analyses for women and men to account for these differences.

This study tested three key hypotheses derived from prior research. Considering the tripartite influence model [7] and subsequent experimental studies [28, 29] as described above, engaging in negative fat talk among women would amplify cognitive processing of thin-ideal body images, activated by seeing slim women on the internet. The increased internalization of the thin ideal may lead to increased body fat dissatisfaction [15]. Therefore, women who engage in negative fat talk more frequently would experience a more substantial indirect effect of exposure to thin-ideal images on body fat dissatisfaction via the internalization of the thin ideal than those who engage in negative fat talk less frequently (Hypothesis 1).^3^ In addition, positive body talk serves as a counterpoint to negative fat talk and is hypothesized to yield opposing effects [31]. Considering this assumption, positive body talk would disrupt cognitive processes that lead to the internalization of the thin ideal triggered by exposure to thin-ideal images on the internet, thereby mitigating increases in body fat dissatisfaction. Therefore, women who engage in positive body talk more frequently experience a weaker indirect effect of exposure to thin-ideal images on body fat dissatisfaction via the internalization of the thin ideal than those who engage in positive body talk less frequently (Hypothesis 2).

Engaging in negative muscle talk may reinforce the internalization of a mesomorphic body ideal due to the shared negative content of negative fat talk and negative muscle talk [1], and because theoretical explanations of negative fat talk may also apply to negative muscle talk [7, 21]. Therefore, we hypothesized that negative muscle talk at baseline would be positively associated with subsequent internalization of athletes’ bodies for women, even after controlling for baseline internalization levels (Hypothesis 3).^4^,^5^

The study also explored the broader influences of body talk types that were not included in the hypotheses, as they were not theoretically justified by the theories discussed above. Such exploratory analyses may provide a more comprehensive understanding of their roles. Furthermore, the study explored whether similar patterns of association among these variables emerged among male participants.^6^,^7^

Examining Hypotheses 1–3 could contribute to expanding the tripartite influence model and its extended versions by incorporating body talk as an active, agentic behavior. Additionally, exposure to traditional media and social media has been identified as a factor that promotes the internalization of idealized body images and body dissatisfaction [17, 19, 20]. This study may clarify whether body talk moderates this relationship. Such an investigation may also further advance research on eating disorder pathology and media psychology.

## Method

### Participants

The study participants were undergraduate and graduate students from Chuo University, International University of Health and Welfare, Meiji Gakuin University, Tokai Gakuin University, Tokushima University, and the University of Tsukuba. Students who agreed to participate completed the study questionnaires in their classrooms after class. A total of 895 individuals completed the questionnaires (Time 1), and 823 participants did so four weeks later (Time 2). Data of participants who did not complete both time points, those who had missing responses for any questionnaire at either time, those who provided inappropriate responses (e.g., identical responses to all items on a questionnaire), or those who did not specify their gender or identified as a gender other than male or female, were excluded from the analyses. The final sample consisted of 386 female students (mean age = 19.22 years, *SD* = 3.24, age range: 18 to 55 years) and 216 male students (mean age = 19.62 years, *SD* = 4.09, age range: 17 to 72 years).^8^ All participants were Japanese except one Canadian, seven Chinese, and one Korean.

Previous longitudinal studies have reported a standard partial regression coefficient of approximately 0.10 for the association between psychological predictors and dependent variables. Therefore, we aimed to recruit a sample of 200 female participants to achieve adequate statistical power to detect a standardized regression coefficient of 0.10 at a significance level of 5%, although this coefficient would not necessarily reach significance under these conditions. As planned, we successfully recruited over 200 female participants, constituting a sufficient sample size for our analysis.^9^ Moreover, we ended up recruiting over 200 male participants, providing an additional sufficiently powered sample for exploratory analysis.

### Measures

#### Body talk scale [1]

The Body Talk Scale was used to assess the frequency of engagement in the three types of body talk: negative fat talk, negative muscle talk, and positive body talk. The scale comprises three subscales, including negative fat talk (5 items), negative muscle talk (4 items), and positive body talk (5 items). Participants rate each item on a 6-point scale ranging from 1 (*never*) to 6 (*always* – *several times per day*). The original and Japanese versions of this scale have demonstrated good validity and adequate to good reliability [1, 24]. At Time 1, the alpha coefficients for the negative fat talk subscale were 0.87 for female and male participants, 0.86 for female and 0.91 for male participants for the negative muscle talk subscale, 0.70 for females and 0.78 for male participants for the positive body talk subscale. At Time 2, the alpha coefficients for the negative fat talk subscale were 0.89 and 0.88 in female and male participants; for the negative muscle talk subscale, they were 0.87 and 0.92, respectively; and for the positive body talk subscale, they were 0.80 and 0.80, respectively.

#### Items assessing exposure to thin-ideal body images on the internet

The authors developed eight items to assess the frequency with which participants viewed or searched for thin ideal body images of individuals of the same sex on the internet. These items assess how often respondents viewed or searched for images and videos depicting: (1) selfie pictures and videos of their acquaintances, (2) fashion models, (3) actors in dramas and movies, (4) individuals exercising in gyms (fitness content), (5) singers and idols, (6) announcers and casters, (7) influencers (e.g., YouTubers and Instagrammers), and (8) athletes. Many individuals depicted in such online content tend to have thin body types, which are widely regarded as ideal.^10^ Participants responded to the prompt, “How often do you view or search for the following images or videos on the internet, including Twitter, Instagram, YouTube, Netflix, or Yahoo!, using your computer or mobile phone?” The online platforms provided as examples in the instructions are commonly used in Japan and thus serve as effective references to help respondents clearly understand what is required.^11^ The participants rated these items on a 6-point scale consisting of 1 (*never*), 2 (*once a month*), 3 (*once in several days*), 4 (*once a day*), 5 (*once every few hours*), and 6 (*once per hour*). At Time 1, the alpha coefficients for these items were 0.86 for both female and male participants. At Time 2, the coefficients were 0.87 for women and 0.85 for men.

#### Sociocultural attitudes towards appearance questionnaire–3 revised [42]

This questionnaire assesses the importance, pressures, social comparisons, and the internalization of appearance ideals. This study used the Japanese version of this questionnaire [43]. Exploratory factor analysis of the Japanese version identified four factors. This study utilized the thin-ideal internalization of people in the media subscales (9 items) and the ideal internalization of athletes’ body subscales (4 items). The thin-ideal internalization of people in the media subscale consists of items from the internalization-TV/magazine (e.g., “I would like my body to look like the people who are on TV”) and internalization-comparison subscales (e.g., “I compare my body to the bodies of TV and movie stars”) in the original scale. The ideal internalization of athlete’s body subscale included items from the internalization-athletes (e.g., “I wish I looked as athletic as sports stars”) and awareness subscales (e.g., “People who have an athletic body are better looking”) in the original scale. The Japanese version has demonstrated good reliability and construct validity among Japanese women and men [43]. Participants rate these items on a 5-point scale ranging from 1 (*definitely disagree*) to 5 (*definitely agree*). Cronbach’s alpha coefficient for the thin-ideal internalization of people in the media subscale was 0.93 at Time 1 and 0.94 at Time 2 for both genders. The alpha coefficients for the ideal internalization of the athlete’s body subscale at Time 1 were 0.77 for women and 0.82 for men, and 0.79 for women and 0.84 for men at Time 2.

#### The body image dissatisfaction scale [44]

This scale, developed based on research conducted with Japanese female participants, assesses an individual’s dissatisfaction related to feeling fat [44]. The scale consists of 24 items, such as “I want to be thinner than I am now” and “Other people think my legs are fat.” This scale has demonstrated good reliability and adequate validity among Japanese women [44] and has been found to be applicable to men, as evidenced by its use in several previous studies involving male participants [45, 46]. Participants rate all items on a 4-point scale ranging from 1 (*not at all true of me*) to 4 (*extremely true of me*). We used the total score to measure body fat dissatisfaction. The alpha coefficients at Time 1 were 0.94 for women and 0.91 for men, and these same values were obtained at Time 2.

This study also used the Japanese Body Silhouette Scale Type-I [47] to measure body fat dissatisfaction among women. However, the results from this scale were very similar to those obtained with the Body Image Dissatisfaction Scale. Therefore, the results from the Japanese Body Silhouette Scale are not reported in the Results section.^12^

#### Demographic data

Participants provided demographic information, including age, gender, university grade, and nationality. To match survey responses across Times 1 and 2, participants also reported their birthdates and the last four digits of their mobile phone numbers.

### Procedure

This study was conducted face to face from May 2023 to August 2023. We recruited participants from their respective universities’ classrooms. Students who voluntarily agreed to participate completed the questionnaires in their classrooms immediately following their lectures. The study received ethical approval from the Ethics Committee of the International University of Health and Welfare and the Ethics Committee of Tokai Gakuin University.

### Statistical analysis

Data from women and men were analyzed separately. Hypotheses 1 to 3 were tested using data from female participants, while data from male participants were analyzed in an exploratory manner. We calculated descriptive statistics and conducted independent samples *t*-tests using SPSS Version 25 (IBM Corporation). All other analyses were conducted using Mplus 8.8 [48]. We performed path analyses with maximum likelihood estimation to evaluate the study hypotheses.

We conducted separate path analyses to test the study hypotheses. We tested the following two models, each including either negative fat talk or positive body talk along with a measure of body dissatisfaction to examine Hypotheses 1 and 2. These models examined the main effects of body talk and exposure to thin-ideal images at Time 1, as well as the interaction between these variables on the internalization of the thin ideal at Time 2, while controlling for the internalization of the thin ideal at Time 1. Additionally, these models examined the main effects of body talk and exposure to thin-ideal images at Time 1, their interaction, and the main effects of the internalization of the thin ideal at Time 2 on body dissatisfaction at Time 2, while controlling for body dissatisfaction at Time 1. We included correlations between exogenous variables. Furthermore, as an exploratory analysis, we examined additional models in which negative muscle talk was substituted for either negative fat talk or positive body talk.

We tested the following model, which included negative muscle talk and a measure of body dissatisfaction, to examine Hypothesis 3. The model assessed the effects of negative muscle talk at Time 1 on the internalization of athletes’ bodies at Time 2, controlling for the internalization at Time 1. It also explored the effects of negative muscle talk at Time 1 and the internalization at Time 2 on body dissatisfaction at Time 2, controlling for baseline body dissatisfaction. Correlations between exogenous variables were included. Furthermore, as an exploratory analysis, we examined alternative models in which negative muscle talk was replaced with negative fat talk or positive body talk.

We assessed the goodness of fit for the models using the Comparative Fit Index (CFI) and the Root Mean Square Error of Approximation (RMSEA), as outlined in the study’s preregistration. According to established guidelines, a CFI greater than 0.90 indicates an acceptable model fit, while a value greater than 0.95 may represent a more stringent criterion [49]. RMSEA values less than 0.05 indicate a close fit, values between 0.05 and 0.08 suggest a reasonable fit, and values greater than 0.10 indicate a poor fit [50].

We addressed missing values using multiple imputations via Bayesian analysis, which was applied separately to the datasets for women and men. For women’s data, the imputation included variables such as age, university, and university grade, as well as all items from the scales used in the study, including the Japanese Body Silhouette Scale Type-I and two unrelated items. The imputation included the same variables for men’s data, excluding scores from the Japanese Body Silhouette Scale Type-I, as we did not administer this measure to men.

## Results

### Descriptive statistics and correlations between variables

Table 1 presents the descriptive statistics for each scale at Times 1 and 2. Table 2 shows the correlations between the measured variables, calculated separately for women and men at the two time points. Independent samples *t*-tests indicated that, at both time points, women compared with men reported significantly higher levels of negative fat talk, exposure to thin-ideal images, the internalization of the thin ideal, and body dissatisfaction. Conversely, men reported significantly higher levels of negative muscle talk, positive body talk, and the internalization of athletes’ bodies (see Table S1).

**Table 1:** Descriptive statistics of study measures

	*n*	*M*	*SD*	Range	Skewness	Kurtosis	*α*
*Women*							
Negative fat talk T1	386	14.92	5.54	5–30	0.19	–0.57	0.87
Negative muscle talk T1	383	8.98	4.17	4–24	0.83	0.29	0.86
Positive body talk T1	383	8.37	3.20	5–22	1.30	2.06	0.70
Exposure to thin-ideal images T1	384	23.02	8.02	8–44	0.11	–0.63	0.86
Internalization of the thin ideal T1	385	30.53	8.94	9–45	–0.59	–0.25	0.93
Internalization of athletes’ bodies T1	386	10.97	3.40	4–20	0.34	–0.01	0.77
Body dissatisfaction T1	384	70.96	14.43	32–96	–0.50	–0.28	0.94
Negative fat talk T2	385	15.38	5.78	5–30	0.27	–0.39	0.89
Negative muscle talk T2	385	9.37	4.28	4–24	0.90	0.74	0.87
Positive body talk T2	385	8.61	3.36	5–22	1.02	0.83	0.80
Exposure to thin-ideal images T2	381	22.71	7.86	8–43	0.03	–0.71	0.87
Internalization of the thin ideal T2	383	29.58	9.30	9–45	–0.44	–0.45	0.94
Internalization of athletes’ bodies T2	384	10.95	3.46	4–20	0.34	–0.05	0.79
Body dissatisfaction T2	380	70.84	14.82	30–96	–0.45	–0.28	0.94
*Men*							
Negative fat talk T1	213	10.18	5.29	5–28	1.07	0.70	0.87
Negative muscle talk T1	213	11.00	5.15	4–24	0.58	–0.30	0.91
Positive body talk T1	215	9.13	3.93	5–26	1.11	1.35	0.78
Exposure to thin-ideal images T1	216	19.55	8.21	8–48	0.51	–0.19	0.86
Internalization of the thin ideal T1	213	23.69	9.64	9–45	0.19	–0.91	0.93
Internalization of athletes’ bodies T1	214	13.40	4.16	4–20	–0.28	–0.66	0.82
Body dissatisfaction T1	215	50.30	13.97	25–93	0.57	–0.06	0.91
Negative fat talk T2	216	10.53	5.78	5–29	1.01	0.36	0.88
Negative muscle talk T2	216	11.40	5.33	4–24	0.63	–0.27	0.92
Positive body talk T2	216	9.59	4.20	5–30	1.53	3.76	0.80
Exposure to thin-ideal images T2	215	20.18	8.01	8–48	0.44	–0.23	0.85
Internalization of the thin ideal T2	215	23.46	10.08	9–45	0.19	–0.92	0.94
Internalization of athletes’ bodies T2	215	13.17	4.40	4–20	–0.19	–0.75	0.84
Body dissatisfaction T2	213	50.97	13.92	24–95	0.57	0.06	0.91

Note: T1 means Time 1, and T2 means Time 2

**Table 2:** Correlations between variables

	1	2	3	4	5	6	7	8	9	10	11	12	13	14
*Time 1*														
1. Negative fat talk T1	–	**0.40**	**0.19**	**0.29**	**0.41**	**0.19**	**0.62**	**0.80**	**0.40**	0.10	**0.26**	**0.38**	**0.17**	**0.58**
2. Negative muscle talk T1	**0.40**	–	**0.29**	**0.28**	**0.21**	**0.37**	**0.17**	**0.32**	**0.73**	**0.20**	**0.22**	**0.18**	**0.38**	**0.13**
3. Positive body talk T1	**0.32**	**0.48**	–	**0.23**	0.06	**0.14**	**–0.14**	**0.12**	**0.18**	**0.62**	**0.13**	0.00	0.09	**–0.17**
4. Exposure to thin-ideal images T1	**0.26**	**0.44**	**0.24**	–	**0.43**	**0.26**	**0.19**	**0.31**	**0.29**	**0.22**	**0.78**	**0.46**	**0.24**	**0.15**
5. Internalization of the thin ideal T1	**0.33**	**0.41**	0.13	**0.44**	–	**0.38**	**0.43**	**0.44**	**0.25**	0.08	**0.40**	**0.80**	**0.34**	**0.43**
6. Internalization of athletes’ bodies T1	**0.28**	**0.52**	**0.13**	**0.31**	**0.62**	–	**0.21**	**0.21**	**0.34**	**0.11**	**0.22**	**0.30**	**0.69**	**0.20**
7. Body dissatisfaction T1	**0.68**	**0.13**	–0.02	0.11	**0.38**	**0.24**	–	**0.60**	**0.24**	–0.06	**0.20**	**0.42**	**0.21**	**0.91**
*Time 2*														
8. Negative fat talk T2	**0.74**	**0.30**	**0.25**	**0.32**	**0.37**	**0.30**	**0.57**	–	**0.51**	**0.21**	**0.35**	**0.47**	**0.25**	**0.62**
9. Negative muscle talk T2	**0.32**	**0.68**	**0.32**	**0.47**	**0.48**	**0.51**	**0.13**	**0.55**	–	**0.31**	**0.29**	**0.26**	**0.40**	**0.22**
10. Positive body talk T2	**0.22**	**0.37**	**0.54**	**0.31**	**0.22**	**0.23**	–0.03	**0.43**	**0.58**	–	**0.20**	0.06	**0.14**	–0.10
11. Exposure to thin-ideal images T2	**0.20**	**0.29**	**0.18**	**0.66**	**0.42**	**0.26**	**0.14**	**0.36**	**0.45**	**0.35**	–	**0.47**	**0.28**	**0.20**
12. Internalization of the thin ideal T2	**0.31**	**0.34**	0.08	**0.40**	**0.73**	**0.47**	**0.32**	**0.42**	**0.47**	**0.22**	**0.50**	–	**0.41**	**0.46**
13. Internalization of athletes’ bodies T2	**0.28**	**0.38**	0.02	**0.25**	**0.48**	**0.64**	**0.17**	**0.29**	**0.45**	**0.20**	**0.32**	**0.61**	–	**0.24**
14. Body dissatisfaction T2	**0.61**	0.09	–0.03	**0.13**	**0.36**	**0.23**	**0.86**	**0.64**	**0.19**	0.01	**0.23**	**0.38**	**0.22**	–

Note: T1 means Time 1, and T2 means Time 2. Data in the upper right side shows correlations in women, and those in the bottom left side shows correlations in men. Correlations in bold are significant at *p* < 0.05

### Path analysis using the internalization of the thin ideal

Table 3 presents the fit indices for separate path analyses using the internalization of the thin ideal at Time 2 as a mediator for women and men. For men, the fit indices of three models using body dissatisfaction as the dependent variable slightly exceeded the threshold for a reasonable approximate fit (RMSEA ≥ 0.08). To improve these models, a path was added from the internalization of the thin ideal at Time 1 to body dissatisfaction at Time 2. However, the results remained unchanged after adding this path (Table S2). Consequently, the original results were adopted.

**Table 3:** Fit indices of models using the internalization of the thin ideal at time 2

	*χ* ^2^	*p*	CFI	RMSEA
*Women*				
Negative fat talk as a body talk scale	2.940	0.086	1.00	0.07
Negative muscle talk as a body talk scale	2.578	0.108	1.00	0.06
Positive body talk as a body talk scale	2.184	0.140	1.00	0.06
*Men*				
Negative fat talk as a body talk scale	2.702	0.100	1.00	0.09
Negative muscle talk as a body talk scale	2.288	0.130	1.00	0.08
Positive body talk as a body talk scale	2.957	0.086	1.00	0.10

**Fig. 1:**
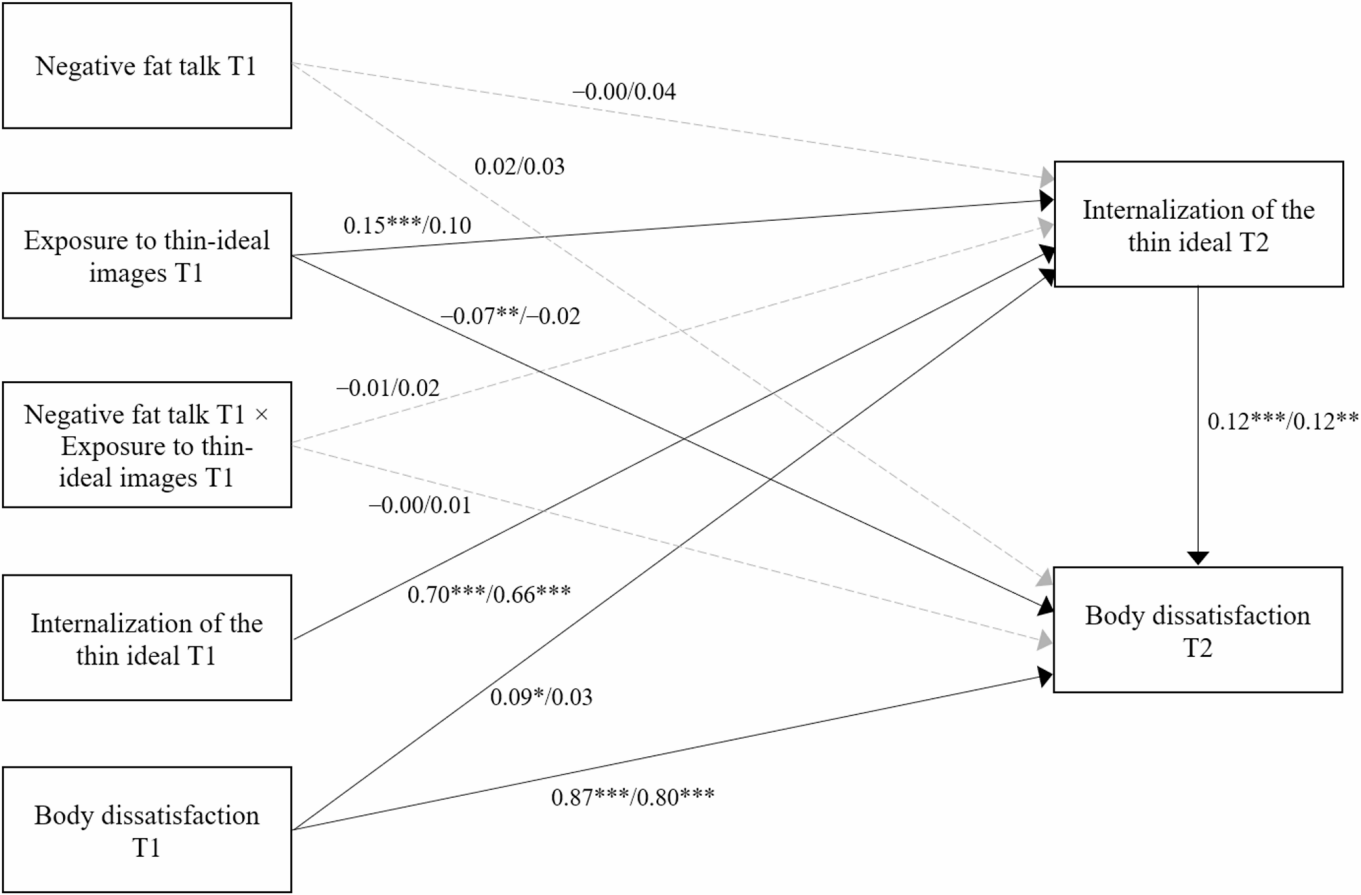
Results of path analyses with the internalization of the thin ideal at Time 2 as a mediator. Values on the left represent results for women, while values on the right represent results for men. Solid lines indicate significant pathways, whereas dotted grey lines indicate pathways that are not statistically significant. T1 means Time 1, and T2 means Time 2

Table 4 presents the results for female participants, with the results of the model using negative fat talk illustrated in Fig. 1. In the hypothesis-testing models including negative fat talk and positive body talk, negative fat talk and positive body talk at Time 1 did not significantly predict the internalization of the thin ideal at Time 2. Similarly, the interactions between negative fat talk and exposure to thin-ideal images, and between positive body talk and exposure to thin-ideal images at Time 1, were not significantly associated with the internalization of the thin ideal at Time 2. However, exposure to thin-ideal images at Time 1 and body dissatisfaction at Time 1 were significantly associated with increased internalization of the thin ideal at Time 2 (see Analyses 1 and 3 in Table 4). An exploratory model using negative muscle talk showed similar patterns: Only exposure to thin-ideal images at Time 1 and body dissatisfaction at Time 1 were significantly associated with increased internalization of the thin ideal at Time 2 (see Analysis 2 in Table 4).

**Table 4:** Women’s path analyses results with the internalization of the thin ideal at time 2 as the mediator

	Internalization of the thin ideal T2				Body dissatisfaction T2		
	*β*	95%CI	*R* ^2^		*β*	95%CI	*R* ^2^
*Analysis 1: Negative fat talk as a body talk scale*			0.67				0.84
Negative fat talk T1	–0.00	[–0.07, 0.07]			0.02	[–0.04, 0.07]	
Exposure to thin-ideal images T1	0.15***	[0.08, 0.21]			–0.07**	[–0.12, –0.02]	
Negative fat talk T1 × Exposure to thin-ideal images T1	–0.01	[–0.07, 0.05]			–0.00	[–0.04, 0.04]	
Internalization of the thin ideal T1	0.70***	[0.63, 0.76]			–	–	
Internalization of the thin ideal T2	–	–			0.12***	[0.07, 0.17]	
Body dissatisfaction T1	0.09*	[0.02, 0.17]			0.87***	[0.83, 0.91]	
*Analysis 2: Negative muscle talk as a body talk scale*			0.67				0.84
Negative muscle talk T1	–0.03	[–0.09, 0.04]			–0.02	[–0.07, 0.03]	
Exposure to thin-ideal images T1	0.15***	[0.08, 0.22]			–0.06*	[–0.11, –0.02]	
Negative muscle talk T1 × Exposure to thin-ideal images T1	0.03	[–0.04, 0.09]			–0.01	[–0.05, 0.04]	
Internalization of the thin ideal T1	0.70***	[0.64, 0.76]			–	–	
Internalization of the thin ideal T2	–	–			0.12***	[0.07, 0.17]	
Body dissatisfaction T1	0.10**	[0.03, 0.16]			0.88***	[0.85, 0.91]	
*Analysis 3: Positive body talk as a body talk scale*			0.67				0.85
Positive body talk T1	–0.06	[–0.13, 0.01]			–0.04	[–0.09, 0.01]	
Exposure to thin-ideal images T1	0.16***	[0.09, 0.23]			–0.06*	[–0.10, –0.01]	
Positive body talk T1 × Exposure to thin-ideal images T1	–0.01	[–0.08, 0.05]			–0.00	[–0.04, 0.04]	
Internalization of the thin ideal T1	0.70***	[0.64, 0.76]			–	–	
Internalization of the thin ideal T2	–	–			0.12***	[0.07, 0.17]	
Body dissatisfaction T1	0.08*	[0.02, 0.15]			0.87***	[0.84, 0.90]	

Note: T1 means Time 1, and T2 means Time 2. Numbers in parentheses indicate 95% confidence intervals

* *p* < 0.05, ** *p* < 0.01, *** *p* < 0.001.

Across all models, none of the body talk types at Time 1 significantly predicted body dissatisfaction at Time 2. In contrast, exposure to thin-ideal images at Time 1 was significantly associated with decreased body dissatisfaction at Time 2. The interactions between each type of body talk and exposure to thin-ideal images at Time 1 were not significantly associated with body dissatisfaction at Time 2. Finally, the internalization of the thin ideal at Time 2 was significantly associated with increased body dissatisfaction at Time 2 across all three models (see Analyses 1–3 in Table 4).

Table 5 presents the exploratory results for male participants, using body dissatisfaction as the dependent variable and applying the same analyses conducted for female participants. None of the body talk at Time 1 was significantly associated with the internalization of the thin ideal at Time 2. Similarly, the interactions between any type of body talk and exposure to thin-ideal images at Time 1 on the internalization of the thin ideal at Time 2 were nonsignificant. Nevertheless, exposure to thin-ideal images at Time 1 was significantly associated with increased internalization of the thin ideal at Time 2 in the model that included positive body talk. Unlike the findings for women, body dissatisfaction at Time 1 was not significantly related to the internalization of the thin ideal at Time 2 across any of the models. The effects of body talk, exposure to thin-ideal images, and their interaction at Time 1 on body dissatisfaction at Time 2 were nonsignificant in all models. Consistent with the results for women, the internalization of the thin ideal at Time 2 was significantly associated with increased body dissatisfaction at Time 2 across all models that included each type of body talk at Time 1. The results of the model using negative fat talk are shown in Fig. 1.

**Table 5:** Men’s path analyses results with the internalization of the thin ideal at time 2 as the mediator

	Internalization of the thin ideal T2				Body dissatisfaction T2		
	*β*	95%CI	*R* ^2^		*β*	95%CI	*R* ^2^
*Analysis 1: Negative fat talk as a body talk scale*			0.54				0.76
Negative fat talk T1	0.04	[–0.11, 0.18]			0.03	[–0.08, 0.14]	
Exposure to thin-ideal images T1	0.10	[–0.01, 0.21]			–0.02	[–0.09, 0.06]	
Negative fat talk T1 × Exposure to thin-ideal images T1	0.02	[–0.07, 0.12]			0.01	[–0.06, 0.09]	
Internalization of the thin ideal T1	0.66***	[0.56, 0.76]			–	–	
Internalization of the thin ideal T2	–	–			0.12**	[0.04, 0.20]	
Body dissatisfaction T1	0.03	[–0.13, 0.19]			0.80***	[0.72, 0.88]	
*Analysis 2: Negative muscle talk as a body talk scale*			0.54				0.76
Negative muscle talk T1	0.03	[–0.10, 0.15]			–0.07	[–0.15, 0.01]	
Exposure to thin-ideal images T1	0.11	[–0.01, 0.23]			0.01	[–0.07, 0.09]	
Negative muscle talk T1 × Exposure to thin-ideal images T1	–0.06	[–0.16, 0.03]			0.03	[–0.05, 0.11]	
Internalization of the thin ideal T1	0.66***	[0.56, 0.76]			–	–	
Internalization of the thin ideal T2	–	–			0.14**	[0.06, 0.22]	
Body dissatisfaction T1	0.06	[–0.05, 0.16]			0.83***	[0.77, 0.88]	
*Analysis 3: Positive body talk as a body talk scale*			0.54				0.76
Positive body talk T1	–0.04	[–0.14, 0.07]			–0.02	[–0.09, 0.05]	
Exposure to thin-ideal images T1	0.12*	[0.00, 0.24]			–0.01	[–0.09, 0.06]	
Positive body talk T1 × Exposure to thin-ideal images T1	–0.06	[–0.17, 0.04]			0.05	[–0.02, 0.13]	
Internalization of the thin ideal T1	0.66***	[0.56, 0.76]			–	–	
Internalization of the thin ideal T2	–	–			0.13**	[0.05, 0.21]	
Body dissatisfaction T1	0.05	[–0.05, 0.16]			0.82***	[0.77, 0.87]	

Note: T1 means Time 1, and T2 means Time 2. Numbers in parentheses indicate 95% confidence intervals

* *p* < 0.05, ** *p* < 0.01, *** *p* < 0.001

### Path analysis using the internalization of athletes’ bodies

Table 6 presents the fit indices for separate path analyses using the internalization of athletes’ bodies at Time 2 as a mediator for women and men. All models met or exceeded acceptable fit criteria.

**Fig. 2:**
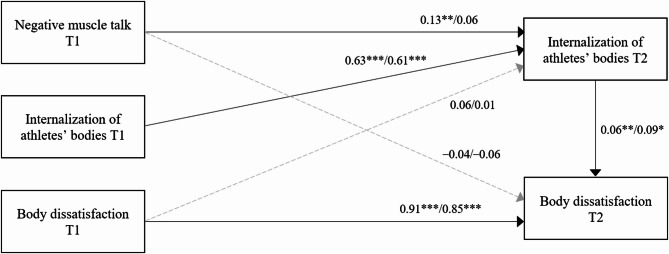
Results of path analyses with the internalization of the athletes’ bodies at Time 2 as a mediator. Values on the left represent results for women, while values on the right represent results for men. Solid lines indicate significant pathways, whereas dotted grey lines indicate pathways that are not statistically significant. T1 means Time 1, and T2 means Time 2

Table 7 displays the results of these analyses for female participants. In the hypothesis-testing model including negative muscle talk, negative muscle talk at Time 1 was significantly associated with increased internalization of athletes’ bodies at Time 2. Body dissatisfaction at Time 1 was not associated with the internalization of athletes’ bodies at Time 2. Additionally, the internalization of athletes’ bodies at Time 2 was significantly associated with increased body dissatisfaction at Time 2, whereas negative muscle talk at Time 1 was not (see Analysis 2 in Table 7). Figure 2 illustrates the results of this model.

Exploratory analyses including negative fat talk and positive body talk at Time 1 indicated that neither variable significantly predicted the internalization of athletes’ bodies at Time 2. Body dissatisfaction at Time 1 was not associated with the internalization of athletes’ bodies at Time 2 in either model. Negative fat talk at Time 1 did not predict body dissatisfaction at Time 2, whereas positive body talk at Time 1 showed a significant negative association with body dissatisfaction at Time 2. Additionally, the internalization of athletes’ bodies at Time 2 was significantly associated with increased body dissatisfaction at Time 2 in both models (see Analyses 1 and 3 in Table 7).

**Table 6:** Fit indices of models using the internalization of athletes’ bodies at time 2

	*χ* ^2^	*p*	CFI	RMSEA
*Women*				
Negative fat talk as a body talk scale	1.934	0.164	1.00	0.05
Negative muscle talk as a body talk scale	1.107	0.293	1.00	0.02
Positive body talk as a body talk scale	1.150	0.284	1.00	0.02
*Men*				
Negative fat talk as a body talk scale	0.869	0.351	1.00	0.00
Negative muscle talk as a body talk scale	0.139	0.709	1.00	0.00
Positive body talk as a body talk scale	0.758	0.384	1.00	0.00

Table 8 presents the exploratory results of path analyses for male participants using body dissatisfaction as the dependent variable, similar to the models for female participants. Negative fat talk at Time 1 was positively associated with the internalization of athletes’ bodies at Time 2. However, neither negative muscle talk nor positive body talk predicted the internalization of athletes’ bodies. Consistent with the results for female participants, body dissatisfaction at Time 1 was not significantly associated with the internalization of athletes’ bodies at Time 2 across any model.

None of the types of body talk at Time 1 were significantly associated with body dissatisfaction at Time 2. However, the internalization of athletes’ bodies at Time 2 was significantly associated with increased body dissatisfaction at Time 2 in models using negative muscle talk and positive body talk at Time 1. In contrast, in the model using negative fat talk, the association between the internalization of athletes’ bodies and body dissatisfaction was nonsignificant. Figure 2 illustrates the results of the model using negative muscle talk.

**Table 7:** Women’s path analyses results with the internalization of athletes’ bodies at time 2 as the mediator

	Internalization of athletes’ bodies T2				Body dissatisfaction T2		
	*β*	95%CI	*R* ^2^		*β*	95%CI	*R* ^2^
*Analysis 1: Negative fat talk as a body talk scale*			0.48				0.84
Negative fat talk T1	0.00	[–0.10, 0.10]			0.02	[–0.03, 0.07]	
Internalization of athletes’ bodies T1	0.68***	[0.61, 0.74]			–	–	
Internalization of athletes’ bodies T2	–	–			0.05*	[0.00, 0.09]	
Body dissatisfaction T1	0.07	[–0.02, 0.16]			0.89***	[0.86, 0.93]	
*Analysis 2: Negative muscle talk as a body talk scale*			0.50				0.84
Negative muscle talk T1	0.13**	[0.05, 0.21]			–0.04	[–0.09, 0.00]	
Internalization of athletes’ bodies T1	0.63***	[0.56, 0.70]			–	–	
Internalization of athletes’ bodies T2	–	–			0.06**	[0.02, 0.11]	
Body dissatisfaction T1	0.06	[–0.02, 0.14]			0.91***	[0.89, 0.93]	
*Analysis 3: Positive body talk as a body talk scale*			0.48				0.84
Positive body talk T1	0.01	[–0.07, 0.08]			–0.05*	[–0.10, –0.00]	
Internalization of athletes’ bodies T1	0.68***	[0.61, 0.74]			–	–	
Internalization of athletes’ bodies T2	–	–			0.05*	[0.01, 0.10]	
Body dissatisfaction T1	0.07	[–0.01, 0.15]			0.90***	[0.87, 0.92]	

Note: T1 means Time 1, and T2 means Time 2. Numbers in parentheses indicate 95% confidence intervals

* *p* < 0.05, ** *p* < 0.01, *** *p* < 0.001

**Table 8:** Men’s path analyses results with the internalization of athletes’ bodies at time 2 as the mediator

	Internalization of athletes’ bodies T2				Body dissatisfaction T2			
	*β*	95%CI	*R²*		*β*		95%CI	*R²*
*Analysis 1: Negative fat talk as a body talk scale*			0.43					0.75
Negative fat talk T1	0.17*	[0.03, 0.31]			0.03		[–0.08, 0.14]	
Internalization of athletes’ bodies T1	0.62***	[0.51, 0.73]			–		–	
Internalization of athletes’ bodies T2	–	–			0.07		[–0.01, 0.14]	
Body dissatisfaction T1	–0.10	[–0.25, 0.06]			0.83***		[0.75, 0.91]	
*Analysis 2: Negative muscle talk as a body talk scale*			0.42					0.75
Negative muscle talk T1	0.06	[–0.05, 0.18]			–0.06		[–0.13, 0.02]	
Internalization of athletes’ bodies T1	0.61***	[0.49, 0.72]			–		–	
Internalization of athletes’ bodies T2	–	–			0.09*		[0.02, 0.17]	
Body dissatisfaction T1	0.01	[–0.09, 0.12]			0.85***		[0.81, 0.90]	
*Analysis 3: Positive body talk as a body talk scale*			0.42					0.75
Positive body talk T1	–0.07	[–0.18, 0.04]			–0.01		[–0.08, 0.06]	
Internalization of athletes’ bodies T1	0.65***	[0.55, 0.75]			–		–	
Internalization of athletes’ bodies T2	–	–			0.07*		[0.00, 0.15]	
Body dissatisfaction T1	0.01	[–0.10, 0.12]			0.85***		[0.81, 0.89]	

Note: T1 means Time 1, and T2 means Time 2. Numbers in parentheses indicate 95% confidence intervals

* *p* < 0.05, ** *p* < 0.01, *** *p* < 0.001

## Discussion

### Predictors and the mediating role of the internalization of the thin ideal

The results of the path analyses for women indicated that neither the main effects of body talk nor the interactions between body talk and exposure to thin-ideal images at Time 1 significantly predicted the internalization of the thin ideal at Time 2 after controlling for the internalization of the thin ideal at Time 1. These results did not support Hypotheses 1 or 2. However, exposure to thin-ideal images at Time 1 was positively associated with the internalization of the thin ideal at Time 2, consistent across the models.

Previous studies have demonstrated that exposure to various media influences the internalization of the thin ideal [19]. Specifically, a meta-analysis by Mingoia et al. [20] revealed that engaging in photo-related activities was more strongly associated with the internalization of the thin ideal than general social networking site use, including sending and receiving private messages. These findings align with the present study, which demonstrated that exposure to thin-ideal images at baseline was associated with an increase in the internalization of the thin ideal four weeks later, even after controlling for baseline levels of the internalization. This result underscores the substantial impact of visual media on the internalization of the thin ideal.

Additionally, the same models revealed no significant association between body talk or its interaction with exposure to thin-ideal images and subsequent internalization of the thin ideal for women. These findings suggest that viewing pictures and videos of slim women exerts a more powerful influence on the internalization of the thin ideal than conversations about one’s weight, whether negative (negative fat talk) or positive (positive body talk). The potent effects of visual stimuli may override any potential influence of body talk on the internalization. This interpretation aligns with the suggestion by Holmes and Mathews [51] that imagery representations have a substantially greater impact on emotional systems and related cognitive and behavioral processes than verbal representations.

Nevertheless, the observed association between exposure to thin-ideal images and increased internalization of the thin ideal could have been artificially strengthened because this study assessed the latter variable using a subscale specifically targeting internalization of thin body shapes portrayed in the media (e.g., “I would like my body to look like the people who are on TV” and “I would like my body to look like the models who appear in magazines”). This specificity may also explain the lack of significant associations between baseline negative fat talk or positive body talk and subsequent internalization of the thin ideal. Future studies should replicate the present findings using a broader measure of the internalization of the thin ideal, such as the Sociocultural Attitudes Towards Appearance Questionnaire-4 [11, 52], which is not limited to media representations of female body shapes.

Mediational analysis revealed that exposure to thin-ideal images was associated with an increase in women’s body dissatisfaction four weeks later via the internalization of the thin ideal. However, exposure to thin-ideal images also negatively affected body dissatisfaction, using the internalization of the thin ideal as a mediator. These contradictory results warrant cautious interpretation, as the simple correlation between exposure to thin-ideal images and body dissatisfaction was positive (see Table 2). This discrepancy may be attributed to the high test-retest correlation of the Body Image Dissatisfaction Scale scores between Time 1 and Time 2. Approximately 83% of the variance in the Body Image Dissatisfaction Scale at Time 2 was explained by its scores at Time 1. This high stability may have introduced a statistical artifact whereby a spurious negative association emerged between exposure to thin-ideal images at baseline and subsequent body dissatisfaction after controlling for the indirect positive association via the internalization of the thin ideal.

For men, the results of path analyses using the internalization of the thin ideal as a mediator mirrored those observed in women. Neither the main effects of body talk nor the interaction between body talk and exposure to thin-ideal images at Time 1 significantly predicted the internalization of the thin ideal at Time 2 after controlling for the internalization of the thin ideal at Time 1. These findings suggest that, in men, body talk does not play a role in the mechanism by which thin-ideal body images are internalized, ultimately leading to increased body fat dissatisfaction. In contrast, path analyses demonstrated that exposure to thin-ideal images at Time 1 positively affected the internalization of the thin ideal at Time 2 in the model including positive body talk, although this effect narrowly missed statistical significance in the models including negative fat talk and negative muscle talk. These results suggest that exposure to thin-ideal images promotes the internalization of the thin ideal across genders.

### Predictors of the internalization of athletes’ bodies

The results of path analyses for women, using the internalization of athletes’ bodies as a mediator, indicated that negative muscle talk at Time 1 was significantly associated with increased internalization of athletes’ bodies at Time 2 after controlling for the internalization of athletes’ bodies at Time 1, which supported Hypothesis 3. The finding for women aligns with predictions based on the extended version of the tripartite influence model, suggesting that women’s engagement in negative muscle talk may promote the internalization of the athletes’ bodies, which corresponds to a toned physique. Furthermore, the internalization of athletes’ bodies was associated with increased body fat dissatisfaction, consistent with research indicating that women also desire a toned and thin muscular body shape [6, 26]. When these desires remain unfulfilled, they may contribute to women’s body dissatisfaction.

Interestingly, for men, baseline negative fat talk—but not negative muscle talk—was associated with increased internalization of athletes’ bodies at Time 2. This finding may suggest that negative fat talk among Japanese men indicates dissatisfaction with a lack of body tone rather than thinness. Athletes in popular Japanese sports, such as soccer and baseball, often have a toned physique. As a result, men who frequently engage in negative fat talk may internalize the ideal of a toned athletic body. In contrast, negative muscle talk, which reflects concerns about muscularity, may not directly relate to the internalizing mesomorphic body commonly admired by Japanese men. These interpretations are consistent with the findings that the internalization of athletes’ bodies was associated with increased body dissatisfaction among men, although this association was only significant in two models using the internalization of athletes’ bodies.

### Limitations

This study has several limitations in addition to the issues discussed above. First, the sample consisted exclusively of Japanese university students. Future research should replicate these findings with other age groups in Japan and populations from different cultural and demographic backgrounds to improve generalizability. Second, this study focused solely on body fat dissatisfaction as a variable and did not include measures of muscle dissatisfaction. Future studies should incorporate both types of body dissatisfaction to provide a more comprehensive understanding of the consequences of internalizing ideal body images.

Third, the items assessing exposure to thin-ideal images on the internet were initially developed by the authors for this study, and their reliability and validity have not been confirmed. In addition, these items provide examples of online platforms commonly used in Japan to view thin-ideal body images; however, they did not include major online platforms such as TikTok and Facebook. Consequently, the range of online platforms participants recalled when responding may have been limited. Furthermore, we assumed that viewing all individuals depicted in the listed images and videos would exert an equivalent influence on the internalization of the thin ideal. However, given that young people tend to use social media more frequently than traditional media, including television and magazines, images and videos of influencers or acquaintances viewed on social media may have a particularly pronounced influence. Future studies should more explicitly specify the online platforms that participants are expected to consider for each item and utilize measures distinguishing between social media and other forms of media.

Fourth, certain variables, such as body dissatisfaction and the internalization of the thin ideal, showed minimal change from Time 1 to Time 2 and were not well explained by other predictors. Future studies may benefit from adopting a longer time interval between measurement points to better capture the potential effects of Time 1 variables on subsequent outcomes. Finally, the study employed a two-wave longitudinal design, which limited the ability to explore causal relationships between variables fully. A design involving three or more time points, combined with analytical approaches such as the random intercept cross-lagged panel model [53], would allow for more robust examinations of causal pathways. We also recommend that future research consider ecological momentary assessment to capture the dynamic associations between state-level body talk, body dissatisfaction, internalizing ideal body images, and recent exposure to ideal images.

## Conclusions

This four-week longitudinal study of Japanese university students demonstrated that neither negative fat talk nor positive body talk moderated the association between exposure to thin-ideal images and the internalization of the thin ideal among women, contradicting Hypotheses 1 and 2. In contrast, exposure to thin-ideal images strongly predicted increased internalization of the thin ideal in women. This internalization was associated with increased body fat dissatisfaction. Notably, exposure to thin-ideal images also emerged as a potential predictor of increased internalization of the thin ideal in men, similar to women.

Additionally, negative muscle talk predicted increased internalization of the athletes’ bodies among women, supporting Hypothesis 3. Among men, negative muscle talk did not significantly predict this internalization, whereas negative fat talk emerged as a significant predictor. The internalization of athletes’ bodies was further associated with increased body fat dissatisfaction in women and men, suggesting that a low-fat, toned, or muscular physique characterizes the internalized ideal athlete’s body across genders.

These findings offer insights into the potential determinants of the internalization of both the thin ideal and athletes’ bodies, as well as body dissatisfaction. Notably, the finding that negative muscle talk facilitates the internalization of athletes’ bodies among women suggests that extending the tripartite influence model by incorporating body talk as an additional predictive factor is appropriate. Furthermore, this study sheds light on mechanisms underlying body dissatisfaction in men, indicating that negative fat talk promotes the internalization of the athletes’ bodies, a pattern that differs from that observed in women.

The present findings highlight the importance of addressing both body talk and exposure to thin-ideal images on the internet to reduce body image concerns. These findings may inform the development of targeted interventions to mitigate these concerns. Further research is needed to refine strategies for improving body image and reducing body dissatisfaction in both women and men.

## Supplementary Information

Below is the link to the electronic supplementary material.

Supplementary Material 1

## Data Availability

This study’s dataset can be found at the Open Science Framework [https://osf.io/z5uas/].

## Footnotes

1 Although there are noticeable differences in muscularity among athletes in sports such as martial arts and weightlifting, researchers often collectively refer to these diverse body shapes as *athletic body shapes*. Consistent with previous research, we employed a general scale that measures the internalization of athletes’ bodies, without differentiating among specific athletic body shapes, to assess the internalization of a mesomorphic ideal. However, because such variations in athletic physiques are important for interpreting our findings, they are discussed in detail in the Discussion section.

2 This article focuses primarily on the social motivations underlying negative fat talk. However, previous studies have also suggested that negative fat talk may represent an expression of body fat dissatisfaction [24].

3 Previous studies have demonstrated that the frequency of exposure to fashion magazines, health and fitness magazines, and posts featuring friends’ fitness-related photos and messages was positively associated with negative fat talk [40, 41]. However, these studies were limited to examining cross-sectional relationships. More importantly, they did not investigate the interactive effects of negative fat talk and exposure to thin-ideal images on the internalization of the thin ideal and body dissatisfaction, which constituted the primary focus of the present study.

4 Given that negative muscle talk was expected to enhance cognitive processes contributing to the internalization of the athletes’ bodies sufficiently, this study examined the primary effect of negative muscle talk on the internalization of the athletes’ bodies. Previous research has predominantly emphasized thin-ideal body images as content on the internet associated with body dissatisfaction, whereas athletic body images have received limited attention [19]. Accordingly, the current study assessed the frequency of exposure to thin-ideal images but not athletic body images. Nevertheless, an interaction between negative muscle talk and exposure to athletic body images may also contribute to the internalization of athletes’ bodies, analogous to the hypothesized interaction between negative fat talk and exposure to thin-ideal images. Future studies should therefore include measures assessing the frequency of exposure to athletic body images.

5 The present study primarily focused on determinants of body fat dissatisfaction (see Ito et al. [24]). To reduce the participants’ burden, the study did not assess muscle dissatisfaction. Future research should investigate the indirect association between negative muscle talk and muscle dissatisfaction, which may occur through the internalization of athletes’ bodies.

6 This study’s hypotheses, methods, and analytical plans were pre-registered (*https://osf.io/z5uas/*).

7 The data analyzed in this study overlap with those used in Ito et al. [24]. However, the previous article did not report findings from analyses involving exposure to thin-ideal images on the internet, the internalization of the thin ideal, or the internalization of athletes’ bodies, which are the primary focus of the present study.

8 The final sample included four women and four men aged 30 years or older, respectively aged 36, 44, 55, and 55 years, and 31, 31, 33, and 72 years. The inclusion of these eight participants is unlikely to significantly affect the results. Therefore, consistent with the pre-registration plan, these participants were retained in the analyses.

9 Although the final size of the female sample was nearly twice the initially planned number of 200, all statistically significant standardized regression coefficients related to our hypotheses exceeded 0.10, suggesting that the observed effects were not only statistically significant but also meaningful in magnitude. Therefore, we believe there is minimal concern regarding overpowered analyses or an inflated risk of detecting trivial effects.

10 We assumed that viewing all individuals depicted in the images and videos included in this item set would equally influence the internalization of the thin ideal. However, given that young people use social media more frequently than traditional media (e.g., television, magazines), the impact of viewing images and videos of influencers or acquaintances on social media may be particularly pronounced, although there is limited clear distinction between individuals featured in traditional media and those featured on social media in Japan. Examining the distinct influences of individuals across different media platforms remains an important area for future research.

11 Although examples of commonly used online platforms in Japan were provided, this may have restricted participants’ recall of other platforms they use. This issue was addressed in the limitations section.

12 The Japanese Body Silhouette Scale Type-I applies only to women, as the silhouette figures included in this scale depict exclusively female bodies [47] (see Ito et al. [24]). Readers interested in these results may contact the corresponding author.
