# Supplementary material for: Body talk and the internalization of the ideal body image: examination based on the tripartite influence model
Source: J Eat Disord. 2025 Nov 13;13:263. doi: 10.1186/s40337-025-01444-2 (PMC12616899; doi:10.1186/s40337-025-01444-2)
Supplement: Supplementary file 1 — Supplementary Material 1 [file 40337_2025_1444_MOESM1_ESM.docx]

**Table S1**

Independent samples t-test results and effect sizes for gender differences in study variables

|  | *df* | *t* | *d* |
| --- | --- | --- | --- |
| Negative fat talk T1 | 597 | 10.20*** | 0.87 |
| Negative muscle talk T1 | 367.07 | –4.91*** | –0.45 |
| Positive body talk T1 | 374.23 | –2.42* | –0.22 |
| Exposure to thin-ideal images T1 | 598 | 5.04*** | 0.43 |
| Internalization of the thin ideal T1 | 410.44 | 8.53*** | 0.74 |
| Internalization of athletes’ bodies T1 | 371.63 | –7.28*** | –0.66 |
| Body dissatisfaction T1 | 597 | 17.00*** | 1.45 |
| Negative fat talk T2 | 599 | 9.87*** | 0.84 |
| Negative muscle talk T2 | 371.39 | –4.78*** | –0.43 |
| Positive body talk T2 | 370.22 | –2.92** | –0.26 |
| Exposure to thin-ideal images T2 | 594 | 3.75*** | 0.32 |
| Internalization of the thin ideal T2 | 596 | 7.49*** | 0.64 |
| Internalization of athletes’ bodies T2 | 362.95 | –6.38*** | –0.58 |
| Body dissatisfaction T2 | 591 | 16.01*** | 1.37 |
| Note: T1 means Time 1, and T2 means Time 2. Degrees of freedom that include decimal values suggest that the result comes from Welch’s *t*-test. A positive *t*-value or Cohen’s *d* suggests that women obtained higher scores, whereas a negative value indicates that men outperformed women. * *p* < 0.05, ** *p* < 0.01, *** *p* < 0.001. | | | |

**Table S2**

Results of path analyses with the internalization of the thin ideal at T2 as the mediator adding path from the internalization of the thin ideal at T1 to the internalization of the thin ideal at T2 for men

|  | Internalization of the thin ideal T2 | | |  | Body dissatisfaction T2 | | |
| --- | --- | --- | --- | --- | --- | --- | --- |
|  | *β* | 95%CI | *R*² |  | *β* | 95%CI | *R*² |
| Analysis 1: Negative fat talk as a body talk scale | | | 0.54 |  |  |  | 0.76 |
| Negative fat talk T1 | 0.04 | [–0.11, 0.18] |  |  | 0.03 | [–0.08, 0.14] |  |
| Exposure to thin-ideal images T1 | 0.10 | [–0.01, 0.21] |  |  | –0.00 | [–0.08, 0.08] |  |
| Negative fat talk T1 ×  Exposure to thin-ideal images T1 | 0.02 | [–0.07, 0.12] |  |  | 0.01 | [–0.07, 0.09] |  |
| Internalization of the thin ideal T1 | 0.66*** | [0.56, 0.76] |  |  | –0.09 | [–0.19, 0.02] |  |
| Internalization of the thin ideal T2 | – | – |  |  | 0.18** | [0.07, 0.29] |  |
| Body dissatisfaction T1 | 0.03 | [–0.13, 0.19] |  |  | 0.82*** | [0.74, 0.90] |  |
|  |  |  |  |  |  |  |  |
| Analysis 2: Negative muscle talk as a body talk scale | | | 0.54 |  |  |  | 0.76 |
| Negative muscle talk T1 | 0.03 | [–0.10, 0.15] |  |  | –0.06 | [–0.14, 0.02] |  |
| Exposure to thin-ideal images T1 | 0.11 | [–0.01, 0.23] |  |  | 0.02 | [–0.06, 0.10] |  |
| Negative muscle talk T1 ×  Exposure to thin-ideal images T1 | –0.06 | [–0.16, 0.03] |  |  | 0.04 | [–0.04, 0.11] |  |
| Internalization of the thin ideal T1 | 0.66*** | [0.56, 0.76] |  |  | –0.08 | [–0.19, 0.03] |  |
| Internalization of the thin ideal T2 | – | – |  |  | 0.19** | [0.08, 0.29] |  |
| Body dissatisfaction T1 | 0.06 | [–0.05, 0.16] |  |  | 0.84*** | [0.78, 0.89] |  |
|  |  |  |  |  |  |  |  |
| Analysis 3: Positive body talk as a body talk scale | | | 0.54 |  |  |  | 0.76 |
| Positive body talk T1 | –0.04 | [–0.14, 0.07] |  |  | –0.01 | [–0.08, 0.06] |  |
| Exposure to thin-ideal images T1 | 0.12* | [0.00, 0.24] |  |  | 0.00 | [–0.07, 0.08] |  |
| Positive body talk T1 ×  Exposure to thin-ideal images T1 | –0.06 | [–0.17, 0.04] |  |  | 0.05 | [–0.02, 0.13] |  |
| Internalization of the thin ideal T1 | 0.66*** | [0.56, 0.76] |  |  | –0.09 | [–0.20, 0.02] |  |
| Internalization of the thin ideal T2 | – | – |  |  | 0.18** | [0.08, 0.29] |  |
| Body dissatisfaction T1 | 0.05 | [–0.05, 0.16] |  |  | 0.84*** | [0.78, 0.89] |  |
| Note: T1 means Time 1, and T2 means Time 2. Numbers in parentheses indicate 95% confidence intervals. There was no test of overall model fit because all models were saturated (i.e., zero degrees of freedom).  * *p* < 0.05, ** *p* < 0.01, *** *p* < 0.001. | | | | | | | |
